# Multi-omics pan-cancer analysis reveals the diagnostic and prognostic value of C8orf76, with experimental validation of its impact on lung adenocarcinoma cell proliferation

**DOI:** 10.3389/fgene.2025.1524422

**Published:** 2025-03-26

**Authors:** Xiaohong Zhong, Zhiyong Zhang, Rongjing Gao, Shiqi Ren, Shifang Li, Miao Zhang, Jie Fang, Yanjiao Hou

**Affiliations:** ^1^ Department of Clinical Laboratory, Qilu Hospital of Shandong University Dezhou Hospital (Dezhou People’s Hospital), Dezhou, Shandong, China; ^2^ Pneumology Department, Qilu Hospital of Shandong University Dezhou Hospital (Dezhou People’s Hospital), Dezhou, Shandong, China

**Keywords:** C8orf76, pan-cancer, multi-omics, cell proliferation, LUAD

## Abstract

**Background:**

Chromosome 8 open reading frame 76 (C8orf76) is a nuclear protein-encoding gene, has received limited attention in current study. Multi-omics pan-cancer analysis focused on the diagnosis, prognosis, immune cell infiltration, methylation, and anti-cancer drug sensitivity remains an enigma. The effect of C8orf76 on lung adenocarcinoma (LUAD) is unknown.

**Methods:**

Multi-omics pan-cancer analysis by utilizing datasets including UALCAN, TIMER 2.0, Human Protein Atlas (HPA), The Cancer Genome Atlas (TCGA), Genotype-Tissue Expression (GTEx), cBioPortal, Gene Expression Profiling Interactive Analysis (GEPIA), OncoDB, and MethSurv datasets, were conducted to analyze C8orf76 across 33 cancer types. Furthermore, differential R packages were uesd for an in-depth analysis of C8orf76. The correlation between C8orf76 expression and diagnostic, prognosis, genetic alteration, mRNA modification, DNA methylation, lncRNA-miRNA-C8orf76 regulatory network, immune cell infiltration, and anti-tumor drugs response were explored to evaluate the potential roles of C8orf76. Most importantly, experiments including quantitative polymerase chain reaction (qPCR), RNA interference (RNAi), Western blotting (WB), and Edu staining, were performed for experimental verification.

**Results:**

It was noted that the C8orf76 expression was markedly elevated across multiple tumor types. Moreover, C8orf76 showed potential as a diagnostic and prognostic biomarker. Besides, it was confirmed that the expression of C8orf76 was related to DNA methylation, mRNA modification, and the infiltration of immune cells. The lncRNA-miRNA-C8orf76 network was established in the study of LUAD. Experimental validation in LUAD A549 cells demonstrated that the knockdown of C8orf76 significantly inhibited cell proliferation in LUAD.

**Conclusion:**

The present study is the first to report that the multi-omics pan-cancer analysis predicts C8orf76 as a promising target in cancer prognosis, diagnosis, immunology, and chemotherapy, highlighting its influence on cell proliferation in LUAD with experimental validation.

## 1 Introduction

Recently, cancer remains a major cause of high morbidity and mortality rates globally, often resulting in substantial health and economic burdens on patients (Chen et al., 2022; Alina and Bernd, 2024; Arielle J et al., 2024; Claudia A J et al., 2024; Claudia G. et al., 2024; Dan et al., 2024). In various cancer types, including gastric cancer (GC) (Guo et al., 2023; Tayah and Jason M, 2024), liver cancer (LIHC), LUAD, and BRCA, early-stage tumors can be surgically removed, including through endoscopic techniques and radical surgery. However, due to the highly aggressive characteristics of most cancers, most patients are diagnosed with advanced cancer, had missed surgery or other treatment opportunities (Muhammad et al., 2024; Nicolas et al., 2024; Nigar et al., 2024), and have poor survival rates (Julia A et al., 2024; Lin et al., 2024; Manel et al., 2024; Moez Karim et al., 2024; Muhammad et al., 2024; Nicolas et al., 2024; Nigar et al., 2024; Ran et al., 2024; Rand et al., 2024). But much less attention has been payed on the new biomarkers which can influence the tumor progression in pan-cancer. Consequently, it is essential to investigate new biomarkers and potential therapeutic targets.

Chromosome 8 open reading frame 76 (C8orf76) refers to a gene that encodes a protein located within the nucleus (Wang and Dong, 2022). Research indicated that C8orf76 plays a role in regulating ferroptosis in liver cancer by enhancing the transcriptional expression of SLC7A11 (Li et al., 2022). Besides, C8orf76 has been demonstrated to enhance the tumorigenic potential and metastatic capabilities of gastric cancer by directly upregulating lncRNA DUSP5P1, and is associated with patient outcomes (Wang et al., 2019). Meanwhile, elevated expression of C8orf76 independently predicted poor prognosis in breast cancer patients (Wang and Dong, 2022). But the relationship between C8orf76 expression and its diagnostic value, genetic alterations, DNA methylation, mRNA modification, and immune cell infiltration in relation to tumor progression remains elusive. Currently, the lack of comprehensive analysis of C8orf76 for pan-cancer is strikingly evident.

To address these gaps, our research utilized a series of comprehensive analysis to elucidate the multifaceted roles of C8orf76 across 33 distinct cancer types, drawing on data from The Cancer Genome Atlas (TCGA) (Jeffery et al., 2018; Jianfang et al., 2018; Tiago Cordeiro and Felipe José Fernández, 2023) and the Genotype-Tissue Expression (GTEx) databases. We conducted analyses of differential mRNA and protein expression, evaluations of diagnostic and prognostic value, assessments of genetic variations, as well as studies on DNA methylation, mRNA modifications, and immune cell infiltration. Furthermore, we constructed lncRNA-miRNA-C8orf76 regulatory network and tried to explore the C8orf76 impact on anti-tumor drug sensitivity.

LUAD, as the prevalent subtype of lung cancer, significantly impacts global mortality rates. Despite advancements in treatment such as surgery, chemotherapy, these methods often do not achieve the desired effectiveness. Therefore, identifying reliable biomarkers that can early predict LUAD remains an urgent need (Yongmei et al., 2018; Xia et al., 2023; Xinqi et al., 2024). In the present study, we wonder if C8orf76 had an effect on cell proliferation in LUAD, to achieve this, we employed a series of experiments in A549 cells, including quantitative polymerase chain reaction (qPCR), RNA interference (RNAi), Western blotting (WB), and Edu staining. Our study represents the inaugural investigation into the capability of C8orf76 as a predictive biomarker for prognosis, diagnosis, and therapeutic interventions in LUAD. Collectively, exploring the broader implications of C8orf76 across various cancer types could provide new insights into its possible function as a universal biomarker for cancer, a target for therapy, and the investigation of its potential clinical applications.

## 2 Materials and methods

### 2.1 Pan-cancer data collection and processing

C8orf76 mRNA expression levels in tumors and normal tissues across different cancer types were analyzed from the TCGA and GTEx datasets. Differential gene expression analyses were conducted using the R “ggplot2” package, and the findings were visualized through radar plots. The bodymap analysis was conducted using the Gene Expression Profiling Interactive Analysis (GEPIA) database (Zefang et al., 2017; Chenwei et al., 2021). Additionally, the UALCAN, HPA, and TIMER2.0 databases were used to analyze the C8orf76 expression levels, which were contrasted between tumor and normal tissues.

### 2.2 Diagnostic and prognostic analysis

The R packages “ggplot2”, “car”, and “stats” were utilized to perform One-way ANOVA analysis to assess the differences in C8orf76 expression among clinical variable groups. The receiver operating characteristic (ROC) curve based on sensitivity and specificity were applied to analyze the diagnostic value of C8orf76 in pan-cancer tissues using the R ‘pROC’ package. Kaplan Meier (KM) curves for C8orf76 were generated utilizing the ‘survminer’ and ‘survival’ packages within the R statistical programming environment.

Univariate Cox regression analyses were subsequently employed to analyze the prognostic relevance of C8orf76 expression as a predictor of OS, DSS, PFI with the R ‘survival’ and ‘forestplot’ packages.

### 2.3 Immunohistochemistry staining and subcellular localization of C8orf76

The Human Protein Atlas (HPA) serves as a comprehensive database for the human proteome, providing insights into the distribution of proteins across various human tissues and cell types. In order to analyze the expression of C8orf76 at the protein level, immunohistochemical images representing various tumor tissues alongside their associated normal tissues were acquired from the HPA database. Additionally, we extracted data pertaining to the structural characteristics and subcellular localization of C8orf76 from the HPA database.

### 2.4 Genetic alteration analysis

cBioPortal offers a comprehensive platform for the analysis and interpretation of genetic data related to cancer, enabling the elucidation of molecular information derived from histological and cytological investigations of cancer. The cBioPortal database was used to obtain the single nucleotide variation (SNV) data across various cancer types. An exploration of the mutation landscape of C8orf76 was conducted, focusing on aspects such as the types of mutations, copy number alterations (CAN), and the frequency of these mutations, utilizing the module known as “Cancer Types Summary.”

### 2.5 Methylation, mRNA modification,and immune cell infiltration analysis

The heatmap analysis of the level of methylation of C8orf76 was obtained from the MethSurv database and OncoDB. MethSurv is an engaging online platform that facilitates both univariate and multivariate survival analyses leveraging DNA methylation biomarkers derived from TCGA data. OncoDB serves as a web-based database resource, enabling researchers to investigate abnormal gene expression patterns and viral infections associated with clinical characteristics in cancer. The analysis of mRNA modifications and immune cell infiltration across various cancers within TCGA was conducted utilizing the R “ggplot2” package.

### 2.6 LncRNA-miRNA-C8orf76 regulatory network construction

Four distinct prediction databases dedicated to miRNAs, including RNA22, DIANA-mircoT, miRcode, and miRDB, were performed identify potential target miRNAs for C8orf76. Additionally, StarBase 2.0 was utilized to investigate the relationship between lncRNAs and miRNAs. The lncRNA-miRNA-C8orf76 interactome network was performed using the Sankey diagram and Cytoscape. Among them, the Sankey diagram was generated using “ggalluvial” and “ggplot2” package implemented in R.

### 2.7 Drugs response analysis

The drug sensitivity assessment of C8orf76 was conducted utilizing CRTP and GDSA datasets. Based on the acquired datasets, FDA-approved chemotherapy drugs linked to C8orf76 expression were identified in drug databases. The results were visualized with Cytoscape tool.

### 2.8 Genes co-expressed with C8orf76 and functional analysis

The analysis identified the top 30 genes showing positive relative co-expression and the top 30 genes demonstrating negative relative co-expression with C8orf76, utilizing R tools to visualize heatmaps based on TCGA data. The PPI network was constructed for the top 10 hub genes displaying positive relative co-expression with C8orf76 using STRING. Further analysis of these hub genes was conducted using “MOCODE” and “CytoHubba” in Cytoscape. Additionally, enrichment analyses including Gene Ontology (GO) (Martha B et al., 2009; Trudy et al., 2009; Paul et al., 2018) and the Kyoto Encyclopedia of Genes and Genomes (KEGG) (Minoru et al., 2022; Minoru et al., 2024), were carried out for the top 300 genes to facilitate cluster information analysis.

### 2.9 Cell lines and cultures

The A549 cell line was sourced from the Chinese Academy of Sciences Cell Bank in Shanghai. The cells were cultured in DMEM medium supplemented with 10% FBS in a 5% CO2 humidified incubator at 37°C.

### 2.10 Quantitative real-time PCR

RNA was extracted by TRlzol. followed by qPCR analysis using SYBR Master Mix (ABclonal, China) on ABI 7500 Real-Time PCR system. Forward primer: CTG​CGA​AGT​CAG​TCC​TTG​C, Reverse primer: CCA​CCT​TCT​TCT​TAC​CTG​TTG​GT. The qPCR cycling conditions included an initial denaturation step at 95°C for 3 min, 40 cycles of denaturation at 95°C for 5 s, annealing at 60°C for 30 s with data collection. The target gene relative expression was assessed using the 2^−ΔΔCT^ method.

### 2.11 RNA interference

The C8orf76-specific siRNA sequence was 5′-GCA​AAU​UGG​CAG​AGG​CUU​ATT-3′, control siRNA sequence was 5′-UUC​UCC​GAA​CGU​GUC​ACG​UTT-3′. Transfection of RNA interference (RNAi) plasmids was conducted using Lip2000 (Invitrogen, United States).

### 2.12 Edu analysis

The cells were treated with Edu (Beyotime, China) for 2 h, followed by the addition of the click reaction solution and subsequent cell incubation. The final results were then analyzed using a fluorescence microscope (Mingmei, China).

### 2.13 Western blotting analysis

Proteins were acquired from A549 cells using a protein extraction kit (Seven, China). The samples were then analyzed by SDS-PAGE, and subsequentlly transferred onto PVDF membranes (Millipore, United States). Following blocking with 5% non-fat milk, the membranes were incubated with primary antibodies (Abcam, United Kingdom) and secondary anti-rabbit antibodies (Abcam, United Kingdom). Finally, protein bands were visualized using chemiluminescence reagents.

### 2.14 Statistical analysis

In this research, the statistical evaluations were performed utilizing the aforementioned online database alongside the R package (R Studio version: 2024.04.2 + 764, R version: 4.3.3), as previously detailed. For the analysis of experimental data, GraphPad Prism 8.0 was employed. Comparisons of differences were carried out using a Student’s t-test, with results expressed as mean ± standard deviation (SD). Statistical significance was determined at *P < 0.05, ***P < 0.001.

## 3 Results

### 3.1 Pan-cancer analyses of C8orf76 expression

**FIGURE 1:**
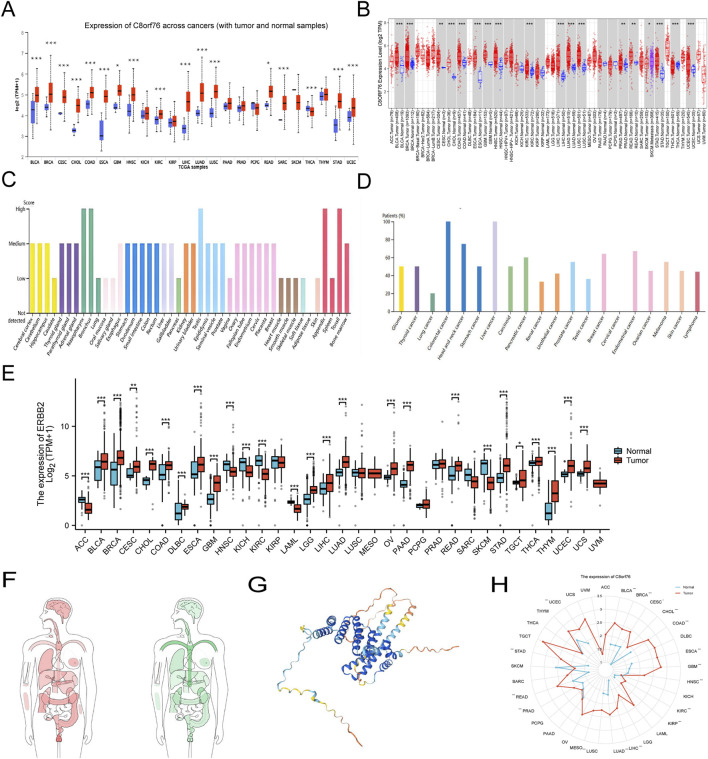
Pan-cancer analyses of C8orf76 expression. **(A)** Analysis of C8orf76 protein levels in primary tumors and normal tissues using UALCAN. **(B)** The TIMER 2.0 database was utilized for comprehensive analysis of C8orf76 expression in both tumor and normal tissues. **(C)** Examination of C8orf76 expression at the protein level among various cancer in the HPA datasets. **(D)** C8orf76 mRNA expression among various cancer in the HPA datasets. **(E)** The TCGA and GTEx datasets were employed for comprehensive analysis of C8orf76 expression in tumor and normal tissues. **(F)** Comparative analysis of C8orf76 expression in human body map, including tumors and normal tissues. **(G)** Molecular structure of C8orf76. **(H)** Radar charts illustrating pan-cancer analyses of C8orf76 in tumors and normal tissues (ns, *P* ≥ 0.05; *P < 0.05; **P < 0.01; ***P < 0.001).

Analysis of the UALCAN database indicated an increase in protein expression of C8orf76 in BRCA, BLCA, CHOL, CESC, COAD, GBM, HNSC, ESCA, KIRC, LIHC, STAD, READ, LUAD, LUSC, SARC, and UCEC, whereas the downregulation of the C8orf76 protein was apparent in THCA (Figure 1A). The relative C8orf76 expression levels across different tumor tissues, obtained from TIMER 2.0 data (Figure 1B). The HPA datasets showed that the C8orf76 levels of protein expression (Figure 1C) and RNA expression (Figure 1D) in different cancer types. The TCGA and GTEx databases were utilized to perform a comprehensive pan-cancer assessment of C8orf76 expression at the mRNA level (Figure 1E). The findings indicated that there was a notable increase in the expression levels of C8orf76 (*P* < 0.001) in BRCA, BLCA, CHOL, COAD, CESC, GBM, DLBC, ESCA, STAD, LGG, OV, LIHC, READ, LUAD, PAAD, THCA, THYM, UCEC and UCS. In contrast, C8orf76 was downregulated (*P* < 0.001) in ACC, HNSC, KICH, KIRC, LAML, SKCM. The body map depicted the comprehensive expression profiles of C8orf76 in different tumor tissues and their respective normal counterparts. Which indicated a significant upregulation of C8orf76 in multiple tumor tissues (Figure 1F, left) compared to the matched normal tissues (Figure 1F, right). Furthermore, as depicted in Figure 1G, we utilized HPA database to investigate the structural characteristics of C8orf76. Additionally, we employed a radar chart to clarify the C8orf76 expression across 33 different tumor types compared with normal tissues, thereby providing a comprehensive overview of its differential expression (Figure 1H).

### 3.2 Immunohistochemical analysis and subcellular localization of C8orf76

**FIGURE 2:**
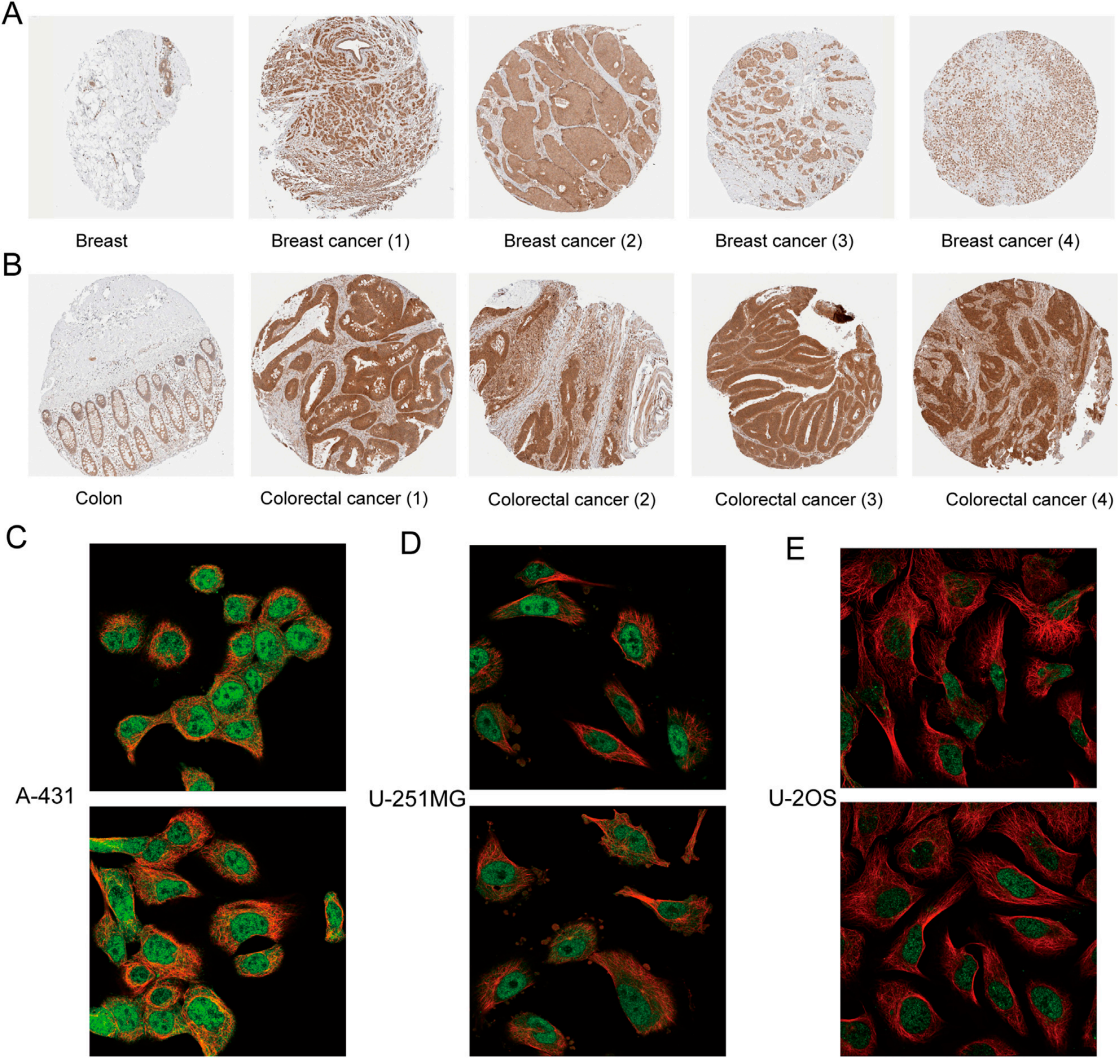
Immunohistochemical analysis and subcellular localization of C8orf76. **(A)** Immunohistochemical expression of protein C8orf76 in breast normal tissues (left) and breast cancer groups (right). **(B)** Immunohistochemical expression of protein C8orf76 in Colon normal tissues (left) and colorectal cancer groups (right). **(C–E)** Subcellular localization of C8orf76 in A-431 cells **(C)**, U-251MG cell line **(D)**, U-2OS cell line **(E)** as determined from the HPA database.

To evaluate the expression of C8orf76 regarding protein levels, we obtained the immunohistochemical images taking advantage of the HPA database. From Figures 2A, B, it is readily apparent that the protein expression of C8orf76 was markedly elevated in breast cancer and colon cancer compared to normal tissues. The subcellular localization of C8orf76 was observed (Figures 2C–E).

### 3.3 C8orf76 offers prognostic and diagnostic value in different cancers

**FIGURE 3:**
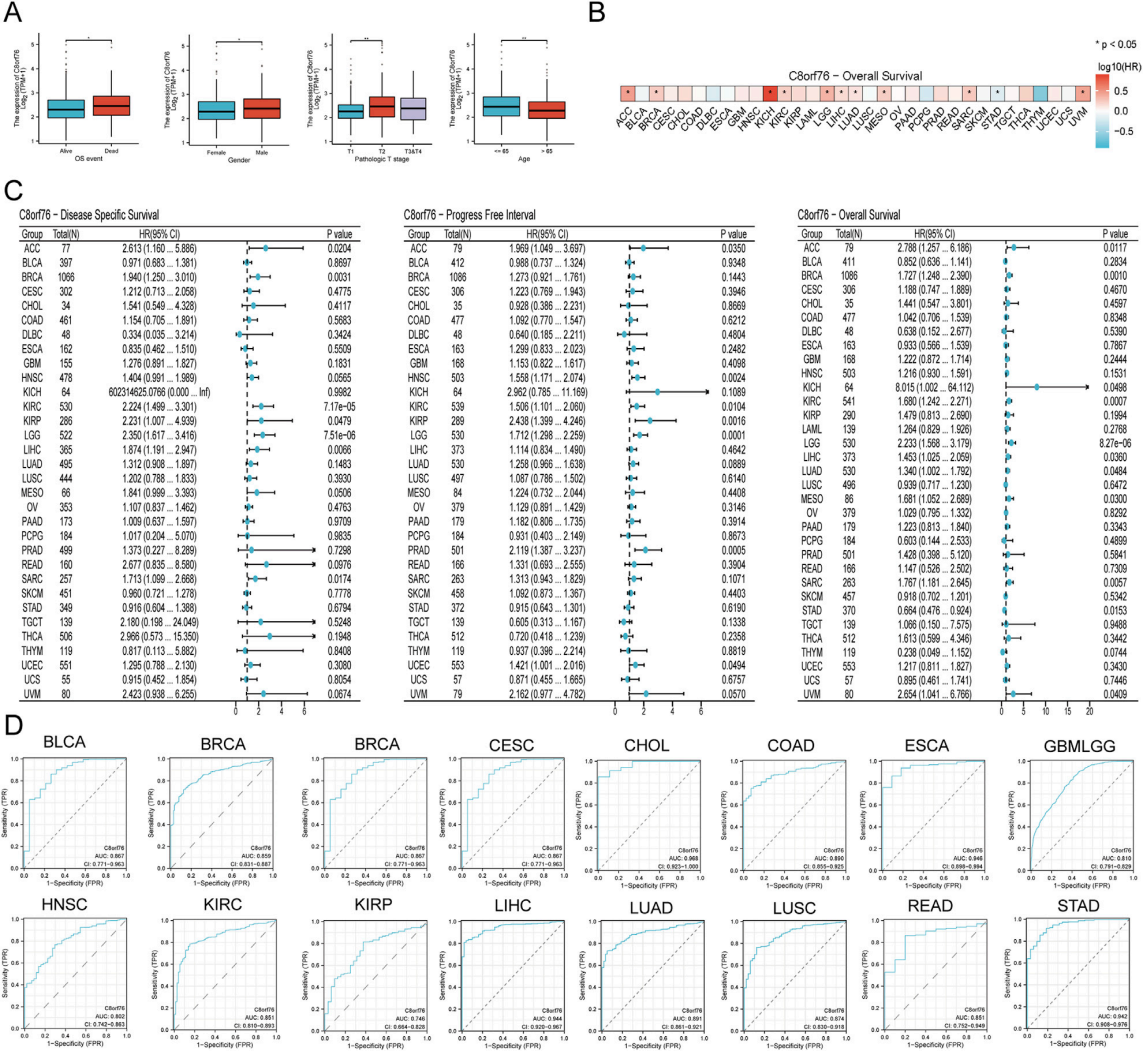
Prognostic and diagnostic analysis in different cancer. **(A)** Examination of the relationship between C8orf76 and clinical data. **(B)** Heat map of C8orf76 gene expression profiles in the pan-cancer with corresponding OS probabilities. **(C)** Forest plots were employed to conduct pan-cancer analyses of C8orf76 in relation to DSS, DFI, and OS. **(D)** AUC of ROC analyses validated the diagnostic potential of C8orf76 across various cancer types.

Analysis revealed a correlation between elevated C8orf76 expression and unfavorable overall survival outcomes in patients. The levels of C8orf76 expression were found to be greater in male patients compared to their female counterparts. C8orf76 expression differs significantly between T1 stage and T2 stage. Significantly higher expression levels of C8orf76 were observed in individuals under the age of 65 compared to those aged 65 and above (Figure 3A). Heat maps were constructed with corresponding OS probabilities (Figure 3B). There were significantly differences in KICH, ACC, LIHC, LGG, BRCA, MESO, KICH, KIRC, LUAD, SARC, STAD, and UVM (Figure 3B).

The prognostic significance of C8orf76 as a predictor of survival outcomes, including DSS, PFI, and OS, was evaluated across different cancer types from TCGA. Univariate Cox regression analyses showed a obvious association between high C8orf76 expression and poorer DSS in ACC, BRCA, KIRP, LIHC, SARC, and THCA. C8orf76 was identified as a significant risk factor linked to worse PFI outcomes in ACC, HNSC, KIRC, KIRP, LGG, and UCEC. With respect to OS, C8orf76 emerged as a prognostic risk factor in ACC, BRCA, KICH, KIRC, LIHC, LUAD, MESO, SARC, STAD, and UVM (Figure 3C).

**FIGURE 4:**
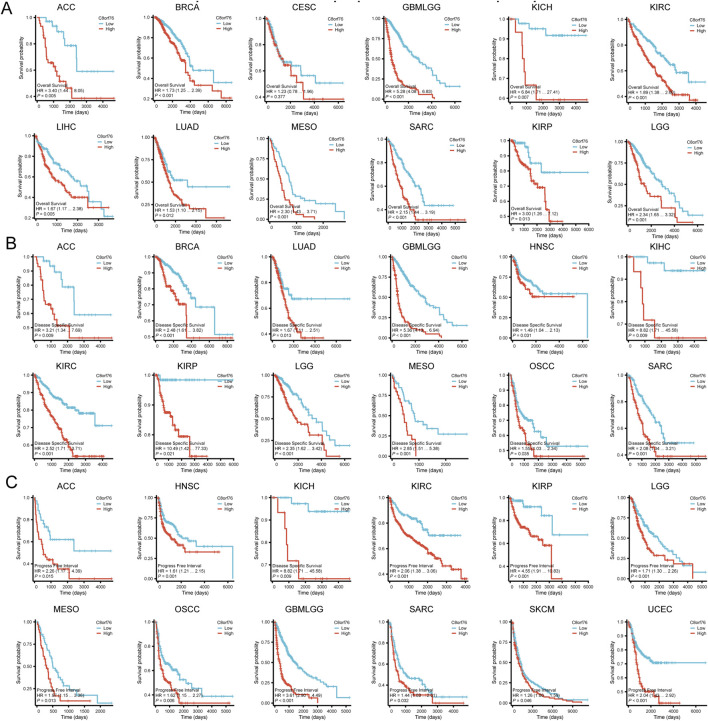
KM analysis of C8orf76 in pan-cancer. These figure presented KM survival analyses demonstrating the correlation between C8orf76 expression and OS **(A)** DSS **(B)** PFI **(C)** across different cancers.

In order to gain deeper insights into the relationship between C8orf76 expression levels and patient outcomes, we conducted Kaplan-Meier (KM) analysis in pan-cancer, focusing on OS, DSS, and PFI. The Cox proportional hazards model analysis revealed that elevated expression levels of C8orf76 were associated with poorer OS in BRCA, CESC, GBMLGG, KIRC, LUAD, MESO, SARC, KIRP, and LGG (*P* < 0.05) (Figure 4A). Moreover, C8orf76 expression levels were identified as a negative prognostic factor for DSS in ACC, BRCA, LUAD, GBMLGG, HNSC, KICH, KIRP, LGG, MESO, OSCC, and SARC (*P <* 0.05) (Figure 4B). Additionally, high C8orf76 expression levels adversely affected PFI in ACC, HNSC, KICH, KIRP, LGG, MESO, OSCC, GBMLGG, SARC, SKCM, and UCEC (*P* < 0.05) (Figure 4C). Figure 3D showed that the Area Under the Curve (AUC) of the Receiver Operating Characteristic (ROC) analysis of the model has high diagnostic accuracy (AUC: 0.9-1.0) in CHOL, ESCA, LIHC, STAD. Relative diagnostic accuracy (AUC: 0.7-0.9) in BRCA, GBMLGG, BLCA, CESC, COAD, READ, HNSC, KIRC, KIRP, LUSC. To sum up, C8orf76 possesses significant prognostic and diagnostic relevance across various cancer types.

### 3.4 Genetic alteration analysis

**FIGURE 5:**
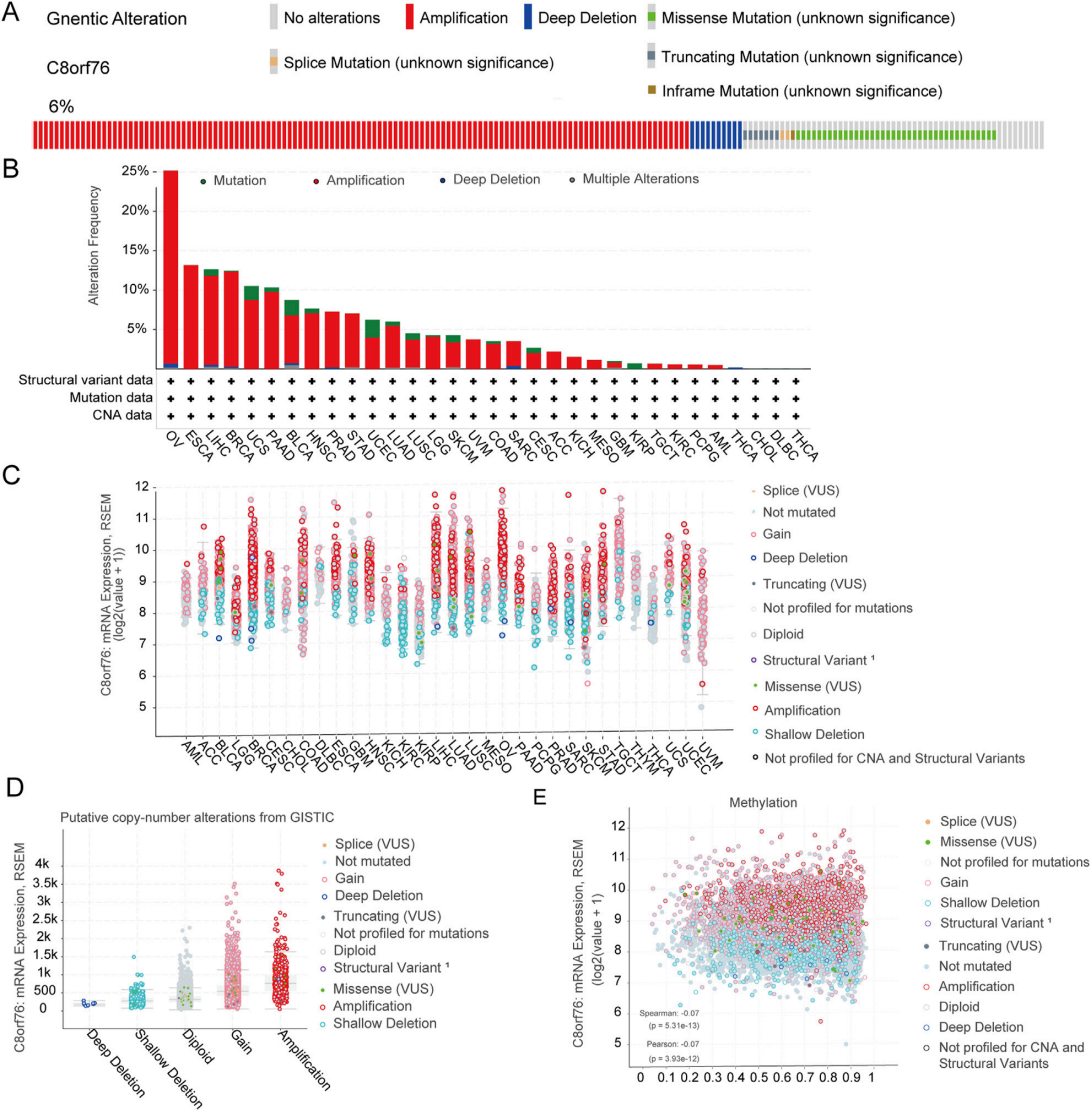
Analysis of Genetic alteration. **(A)** The prevalence of genetic modifications in C8orf76 across various pan-cancer tissues, representing 6% of total alterations. **(B)** The frequency of alterations categorized by mutation type for C8orf76 in diverse cancer types. **(C)** The mRNA expression levels of C8orf76 in relation to its putative CNA across pan-cancer tissues. **(D)** The mRNA expression of C8orf76 putative CNA from GISTIC. **(E)** The mRNA expression of C8orf76 methylation.

We conducted analysis of the genetic modifications of C8orf76 across various cancer types utilizing cBioPortal. There were notable alterations in the expression of C8orf76 within pan-cancer tissues, which accounted for 6% of the samples, as illustrated in Figure 5A. The frequencies of these mutations differ among different cancer types. In OV, elevated mutation frequencies have been detected (Figure 5B). The predominant alterations identified in pan-cancer tissues were classified as the “amplification” and “gain” types in copy-number alteration (CNA) (Figure 5C). Subsequently, as illustrated in Figure 5D, we examined the relationship between methylation of C8orf76 and putative CNA from GISTIC. Subsequently, an assessment of the mRNA expression associated with the methylation status of C8orf76 was performed (Figure 5E).

### 3.5 Methylation analysis of C8orf76

**FIGURE 6:**
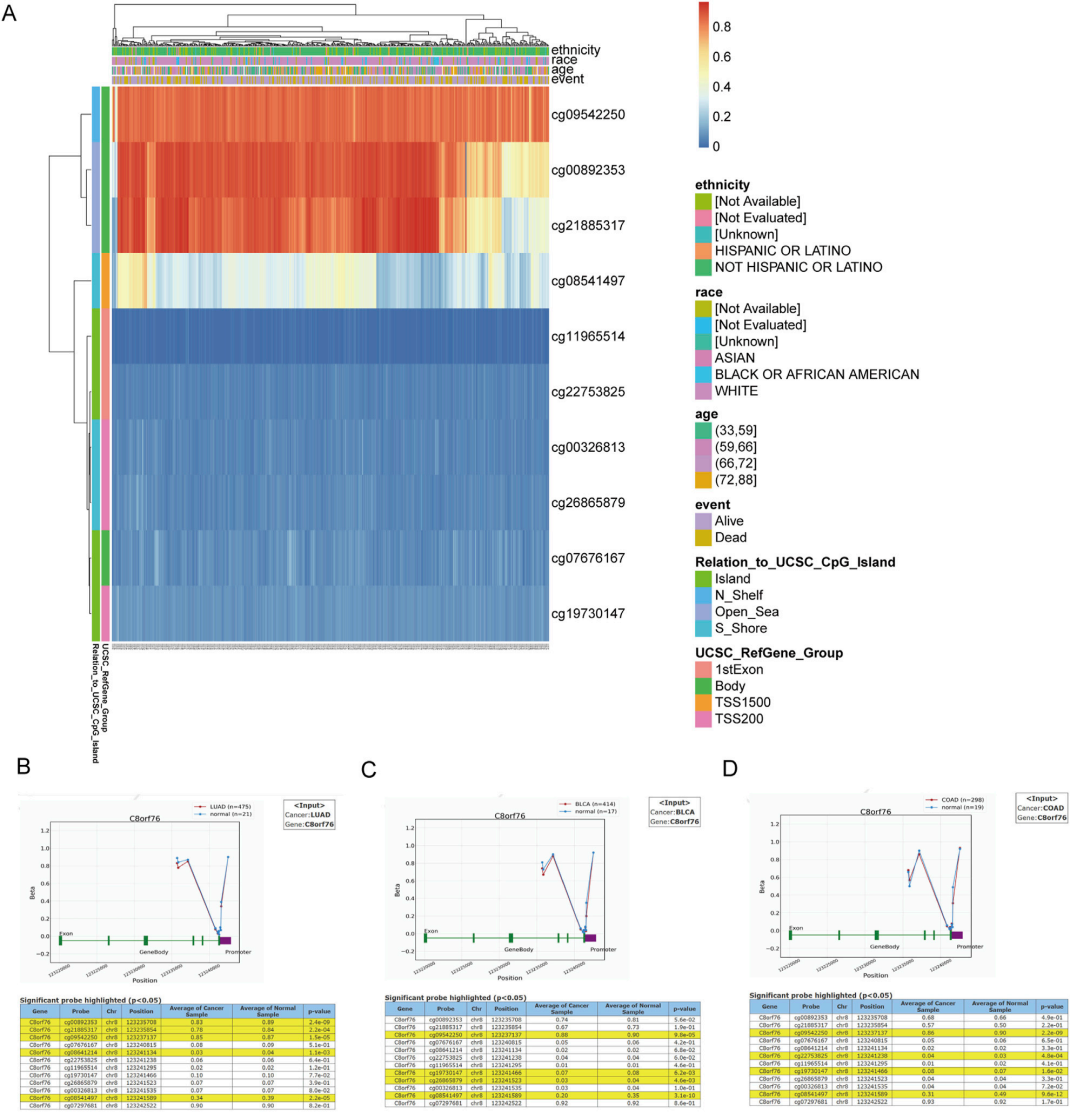
Methylation analysis of C8orf76. **(A)** The heatmap of C8orf76 DNA methylation in LUAD. Exploration of C8orf76 methylation status in LUAD **(B)** BLCA **(C)** and COAD **(D)** was performed using the OncoDB database.

To investigate the DNA methylation levels of the C8orf76 gene, gene expression data derived from the MethSurv database were utilized (Figure 6A). To enhance the understanding of the macroscopic dysregulation of methylation expression by examining microscopic variations at specific methylation sites, we performed additional validation analysis utilizing the OncoDB database. Our findings revealed that, relative to adjacent normal tissues, LUAD demonstrated high methylation levels at the cg00892353, cg21885317, cg09542250, cg08641214 and cg08541497 sites, BLCA showed elevated methylation at cg09542250, cg19730147, cg26865879, and cg08541497. COAD displayed increased methylation at cg09542250, cg22753825, cg19730147,and cg08541497 (Figures 6B–D).

### 3.6 C8orf76 expression and mRNA modification

**FIGURE 7:**
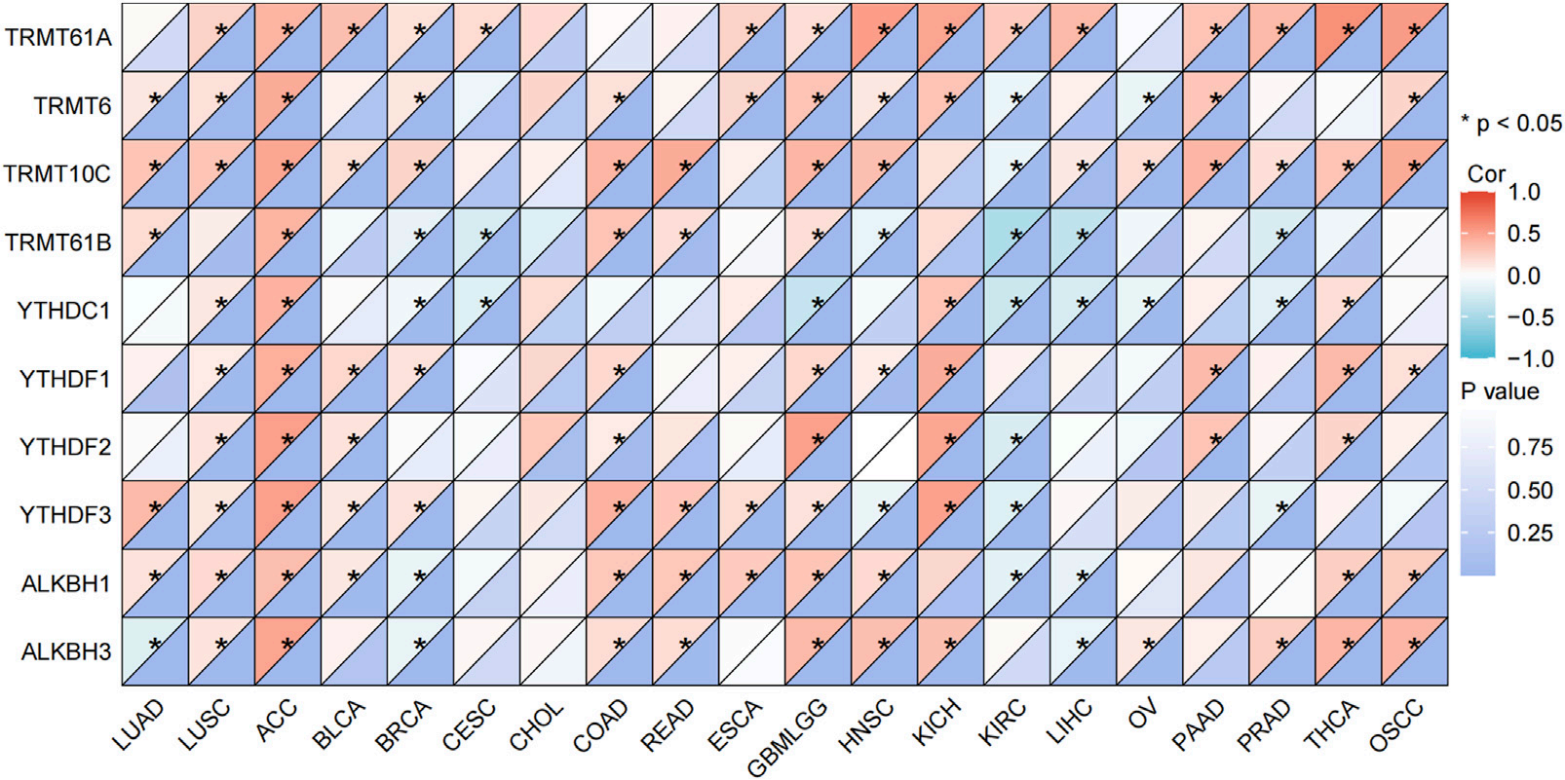
Correlation analysis of the relationship between the C8orf76 expression levels and the regulatory factors involved in mRNA modification methylation across various cancer types.

As shown in Figure 7, C8orf76 expression was positively related to several regulatory factors involved in the methylation of mRNA modifications across various cancer types, included YTHDF3 (r = 0.364, P < 0.001), TRMT10C (r = 0.308, P < 0.0001) in LUAD; TRMT61A (r = 0.400, P < 0.001), TRMT61 (r = 0.444, P < 0.0001), TRMT10C (r = 0.476, P < 0.0001), TRMT61B (r = 0.401, P < 0.001), YTHDC1 (r = 0.409, P < 0.001), YTHDF2 (r = 0.513, P < 0.0001), YTHDF3 (r = 0.511, P < 0.0001), ALKBH1 (r = 0.482, P < 0.01), ALKBH3 (r = 0.482, P < 0.0001) in ACC; TRMT61A (r = 0.338, P < 0.0001) in BLCA; YTHDF3 (r = 0.407, P < 0.0001) in COAD, YTHDF2 (r = 0.474, P < 0.0001), TRMT61A (r = 0.477, P < 0.001) in KICH; TRMT61A (r = 0.594, P < 0.0001) in THCA. The results indicated that the expression levels of C8orf76 are significantly linked to DNA methylation (Figure 6) and mRNA modification (Figure 7) across various types of cancer, including LUAD.

### 3.7 Immune cell infiltration analysis

**FIGURE 8:**
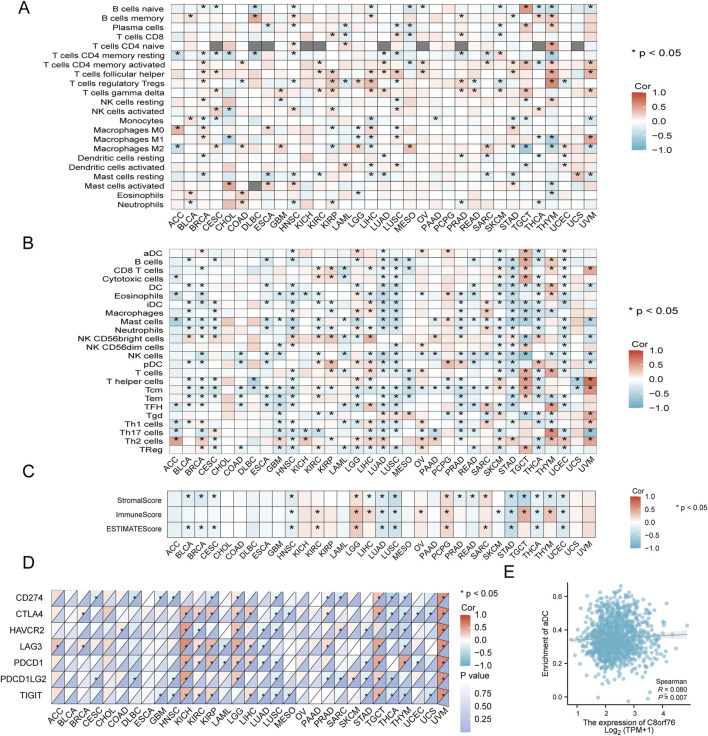
Analysis of the relationship between C8orf76 expression and the immune cell infiltration. **(A) **The associations between the levels of C8orf76 and the infiltration of immune cells as estimated by the CIBERSORT algorithm across various cancer types. **(B)** Analysis of the associations between C8orf76 expression and immune cell infiltration in various cancer, assessed using the ssGSEA algorithm. **(C)** A heatmap illustrating the correlation between C8orf76 expression and immune infiltration was generated using ESTIMATE, Immune, and Stromal scores. **(D)** The relationship between the expression levels of C8orf76 and ICP genes expression. **(E)** The associations between C8orf76 mRNA expression and the enrichment of aDC in BRCA.

We conducted an analysis to explore the correlation between the expression levels of C8orf76 and the infiltration of immune cells in the tumor microenvironment (TME) by various algorithms. The results from CIBERSORT revealed that the C8orf76 expression was associated with cancer-associated immune cells in different cancers, particularly in BRCA, LIHC, KIRP, HNSC, LUSC, LUAD, THYM, and TGCT. The expression levels of C8orf76 exhibited a positive association with the presence of infiltrating regulatory T cells (Tregs), follicular helper T cells, and CD4 naive T cells in THYM; with M1 macrophages in UVM; with naive B cells in TGCT; with activated mast cells in CHOL; and with M0 macrophages in ACC (Figure 8A). The results from ssGSEA further indicated that C8orf76 expression was linked to cancer-associated immune cells across various cancers, especially in LUAD, LUSC, SKCM, TGCT, THCA, THYM, HNSC, UCEC, and LGG. C8orf76 expression levels positively correlated with T cells, T helper cells, Tcm, TReg, aDC, B cells, Cytotoxic cells, TReg in TGCT; with T helper cells, Tcm, Th2 cells, and CD8 T cells in UVM; with activated mast cells in CHOL; with Th17 cells, TFH, T cells in THYM, with Th2 cells in ACC, and with NK CD56 bright cells in LIHC (Figure 8B). Subsequently, the ESTIMATE algorithm was used to assess the relationship between C8orf76 and various immunological features across 33 different tumor types. A significant positive correlation was observed between the ESTIMATE scores and the Immune score values specifically for LGG, PCPG, and SARC, the opposite was evident for HNSC, STAD, UCEC, LUAD, LUSC (Figure 8C).

The immune checkpoint (ICP) gene has been identified as a significant factor influencing the infiltration of immune cells and the effectiveness of immunotherapy. Our findings revealed a positive correlation between C8orf76 expression levels and PDCD1, HAVCR2, CD274, LAG3, CTLA4, PDCD1LG2, and TIGIT in UVM; with CTLA4, PDCD1, LAG3, HAVCR2, PDCD1LG2, and TIGIT in KIHC; and with CTLA4, TIGIT, PDCD1, PDCD1LG2, CD274, and LAG3 in TGCT (Figure 8D). The results revealed that C8orf76 expression was positively related to the enrichment of aDC in BRCA (Figure 8E).

Immune checkpoint (ICP) genes have been identified as significant factors influencing the infiltration of immune cells and the effectiveness of immunotherapy. Our findings revealed a positive correlation between the expression levels of C8orf76 and CD274.

### 3.8 Construction of the lncRNA-miRNA-C8orf76 regulatory network in LUAD.

**FIGURE 9:**
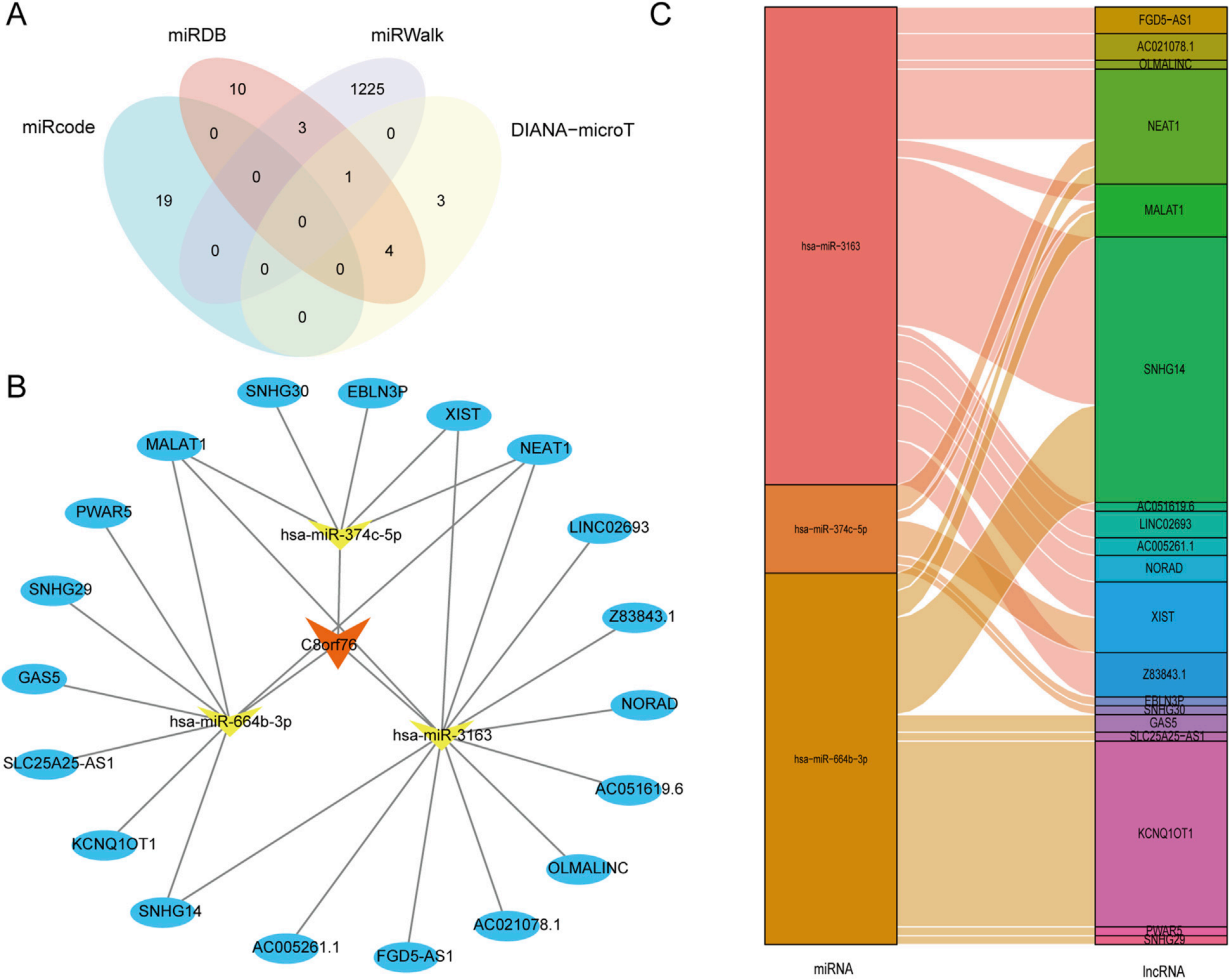
Construction of a crucial ceRNA network associated with C8orf76 in LUAD. **(A)** A Venn diagram showing the predicted target miRNAs of C8orf76 obtained from miRcode, DIANA-microT, miRWalk, and miRDB databases. **(B)** The regulatory network involving lncRNA-miRNA-C8orf76 was developed for LUAD using Cytoscape tools. **(C)** Sankey diagram of target miRNAs and their target lncRNAs.

The databases miRcode, DIANA-mircoT, miRWalk, and miRDB were performed to determine the target miRNAs associated with C8orf76 in LUAD, as illustrated in Figure 9A. From these resources, we successfully identified 19, 10, 1225, and 3 target miRNAs corresponding to C8orf76, respectively. Following this, we developed a regulatory network involving lncRNAs-miRNAs-C8orf76 specific to LUAD, depicted in Figure 9B. Additionally, we conducted a Sankey diagram to show three target miRNAs and their corresponding target lncRNAs (Figure 9C).

### 3.9 Sensitivity of C8orf76-related drugs across multiple cancer types

**FIGURE 10:**
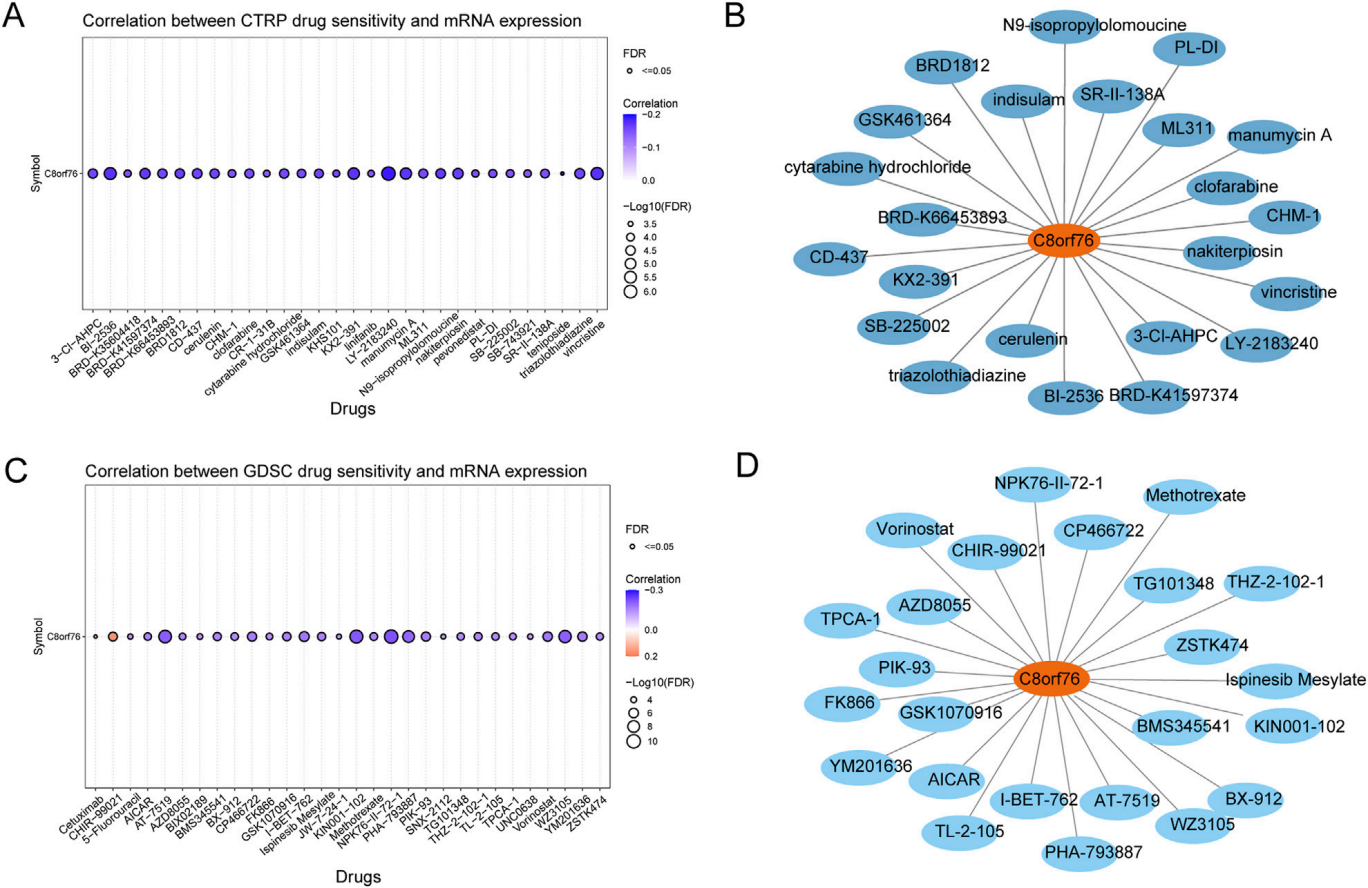
Sensitivity of drugs associated with C8orf76 across various pan-cancer tissues. **(A)** The correlation between CTRP drug sensitivity and C8orf76 mRNA expression. **(B)** FDA-approved chemotherapeutic agents related to C8orf76 identified through the CTRP drug sensitivity analysis. **(C)** The relationship between drug sensitivity from the GDSC and C8orf76 mRNA expression levels. **(D)** FDA-approved anti-cancer drugs associated with C8orf76 derived from based on GDSC drug sensitivity results.

The CTRP database revealed the associations between the expression levels of C8orf76 mRNA and drug sensitivity. An inverse relationship suggests that increased levels of C8orf76 may enhance drug sensitivity, whereas a positive correlation indicates that elevated levels of this gene could contribute to drug resistance. The findings demonstrated that LY−2183240, BI−2536, vincristine, and KX2−391 were negatively correlated with C8orf76 expression (Figure 10A). Additionally, analyses based on drug sensitivity data from the GDSC indicated that CHIR−99021 and Cetuximab exhibited a positive correlation with C8orf76 expression, while NPK76−II−72−1, KIN001−102, and Vorinostat showed a negative correlation with C8orf76 expression (Figure 10C).

Some of the drugs identified in the predicted outcomes for drug sensitivity assessments from CTRP and GDSC databases are utilized in the realm of scientific investigation. Specifically, a total of 23 antitumor drugs associated with C8orf76 were predicted using the CTRP dataset, which were FDA-approved, were derived from data sourced from drug banks (Figure 10B). Additionally, 24 types of C8orf76-related antitumor drugs, also FDA-approved and predicted by the GDSC datasets, were similarly informed by drug bank data (Figure 10D).

### 3.10 Analysis of co-expression and functional enrichment associated with C8orf76 expression in LUAD

**FIGURE 11:**
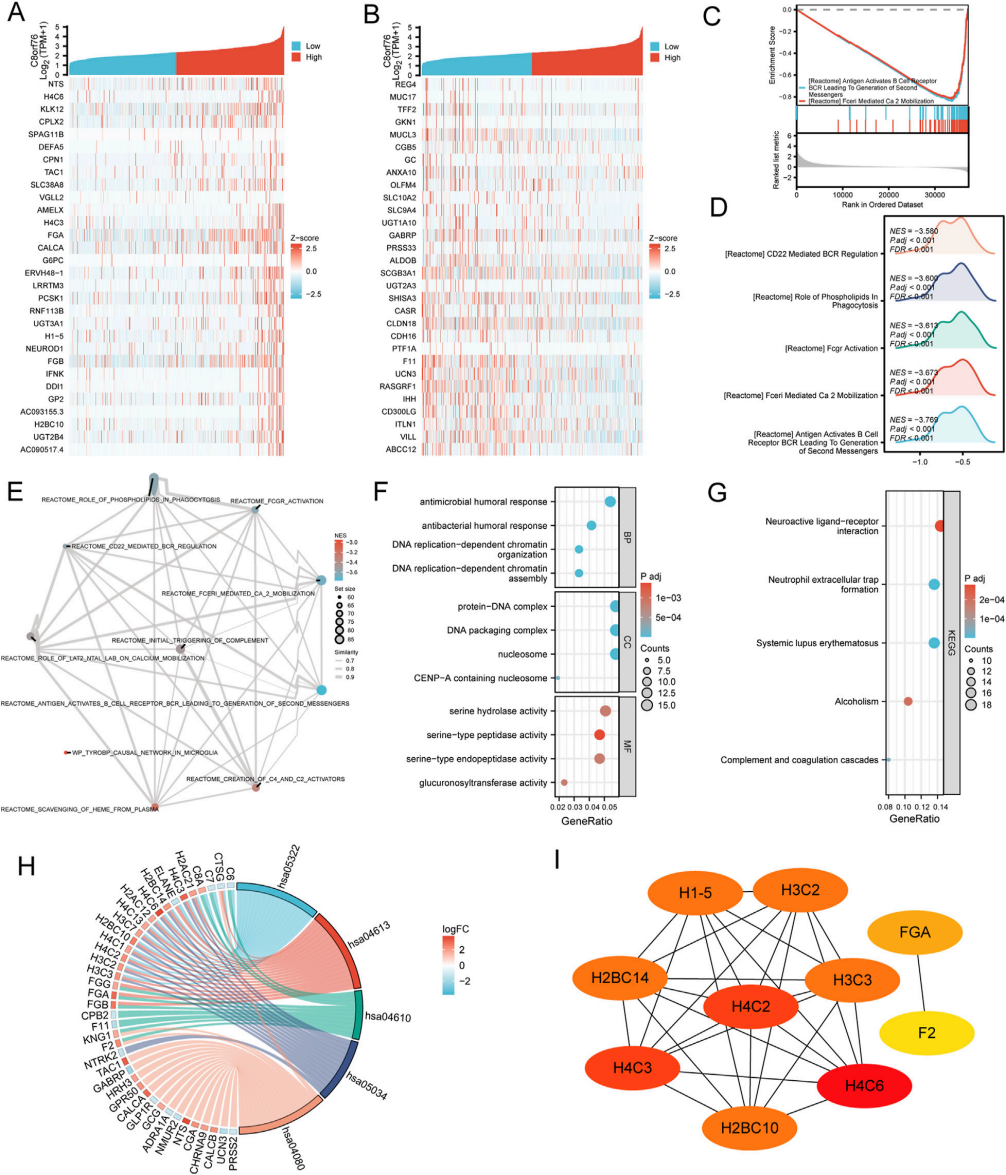
Analysis of co-expression and functional enrichment associated with C8orf76 expression in LUAD. **(A, B)** Heatmaps displaying the expression levels of the top 30 genes positively correlated with C8orf76 expression and the top 30 genes negatively correlated with C8orf76 expression in LUAD. **(C–E)** Functional enrichment analysis was conducted with the “classic”,“mountain plot”,“EMAP” GSEA. **(F, G)** GO and KEGG analysis of C8orf76 and the top 300 co-expressed genes. **(H)** The visualization result of a combined GO KEGG enrichment analysis for the top 300 co-expressed genes based on log2 fold change (logFC) is presented as a chord diagram. **(I)** Identification of the top 10 hub genes in the network and the MCODE2 components.

We discovered genes that revealing co-expression patterns using data from TCGA. Results showed the top 30 co-expressed upregulated genes and downregulated genes correlated with C8orf76 expression in LUAD by the heatmap (Figures 11A, B). The Gene Set Enrichment Analysis (GSEA) analysis was presented in three forms: classical plot (Figure 11C), mountain plot (Figure 11D), and EMAP plot (Figure 11E). Subsequently, KEGG and GO enrichment analyses were performed on the top 300 co-expressed genes. GO analysis revealed that these genes were highly enriched for functions related to antimicrobial humoral response, DNA replication-dependent chromatin organization, serine-type peptidase activity, serine hydrolase activity (Figures 11F, G). The findings of the KEGG pathway enrichment analysis indicated that C8orf76 expression played an important role in LUAD through neuroactive ligand receptor interactions. The visualization results of the combined GOKEGG enrichment analysis for the top 300 genes based on log2 fold change (logFC) are presented using a chord diagram (Figure 11H). The top 10 hub genes were H4C2, H3C3, H2BC14, H3C2, H1-5, H4C3, H2BC10, H4C6, FGA and F2 (Figure 11I).

### 3.11 Investigation of immune cell infiltration associated with C8orf76 expression in LUAD

**FIGURE 12:**
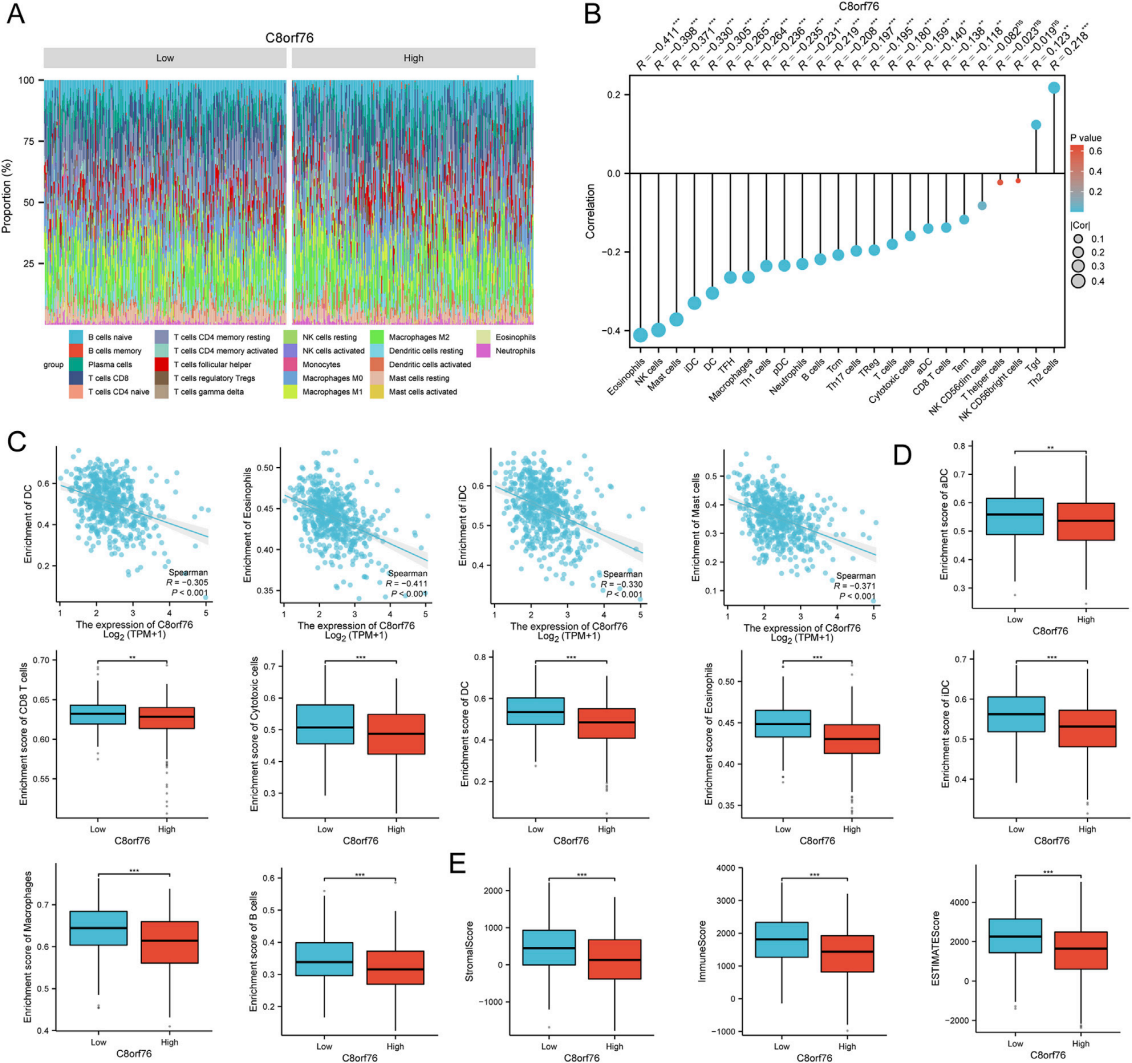
Investigation results of immune cell infiltration associated with C8orf76 expression in LUAD. **(A)** Comparison of immune cell infiltration results between high and low expression groups of C8orf76 in LUAD, a superimposed bar graph was used to show the distribution of immune cell infiltration. **(B)** The correlation between C8orf76 and immune cell infiltration was performed in LUAD and the results were presented in lollipop chart form. **(C)** Scatter plot analysis was conducted to assess the relationship between C8orf76 expression and immune cell infiltration in LUAD. **(D)** The ssGSEA algorithm was performed to investigate the association between high and low expression groups of C8orf76 and immune cell infiltration outcomes in LUAD. The findings were visualized in a bar chart. **(E)** The ESTIMATE algorithm was performed to investigate the association between high and low expression groups of C8orf76 and immune cell infiltration outcomes in LUAD. The findings were visualized in a bar chart. *P < 0.05, ***P < 0.001.

A comprehensive analysis was performed to assess the correlation between immune cell infiltration and C8orf76 expression in both high and low expression groups in LUAD. The results are presented in the form of superimposed bar chart (Figure 12A), lollipop chart (Figure 12B), scatter chart (Figure 12C) and bar graph (Figures 12D, E) respectively. The results demonstrated that C8orf76 expression was negatively related to Eosinophils, Mast cells, iDC cells and DC cells (Figures 12B, C) and NK cells (Figure 12B). CD8 T cells, B cells, Cytotoxic cells, aDC cells, Eosinophils, Macrophages, iDC cells and DC cells enrichment scores were lower in the group with high expression of C8orf76 by ssGSEA algorithm (Figure 12D). The group with high expression of C8orf76 exhibited lower immune scores and stromal scores as calculated by the ESTIMATE algorithm (Figure 12E).

**FIGURE 13:**
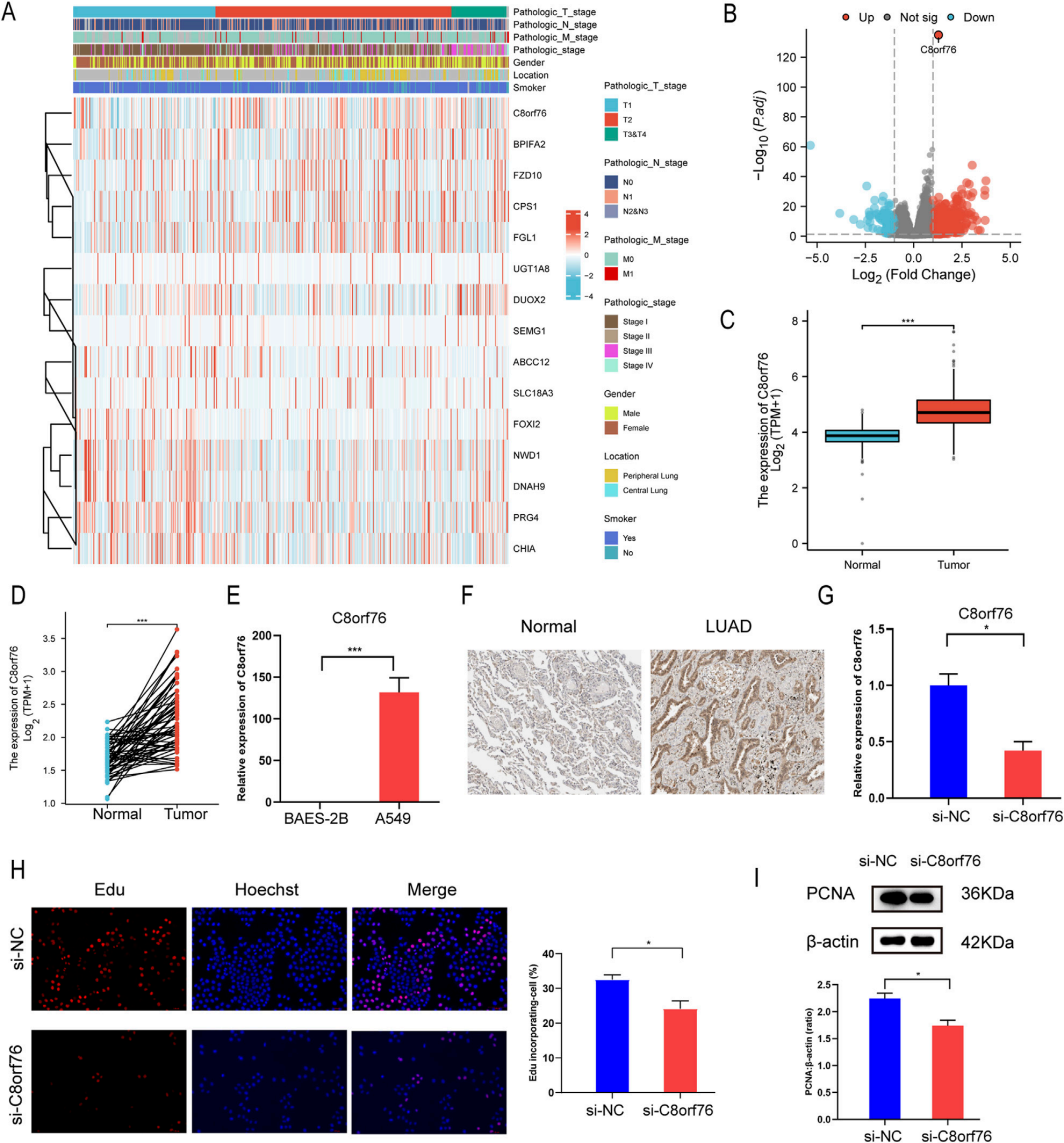
Knockdown of the C8orf76 gene can suppress the proliferation of A549 LUAD cells. **(A)** Visual analysis of heat maps related to C8orf76 and clinical variables was performed using ComplexHeatmap package. **(B)** Volcano graph of mRNA differential analysis results in LUAD. **(C)** Analysis of C8orf76 expression levels in LUAD and matched normal tissues derived from TCGA. **(D)** Unpaired analyses C8orf76 expression in TCGA LUAD and normal tissues. **(E)** qPCR was performed to evaluate the expression levels of C8orf76 in A549 LUAD cells, in comparison to human normal lung epithelial cells (BEAS-2B). **(F)** Immunohistochemical detection of C8orf76 in normal lung tissues and LUAD tissues from HPA database. **(G)** qPCR analysis was conducted to analyze the effectiveness of C8orf76 knockdown in A549 cells. **(H)** Analysis of proliferation in A549 cells with silenced C8orf76 and control groups using Edu immunofluorescence staining. Red and blue staining indicate Edu and Hoechst nuclear staining, respectively. Scale bar: 100 μm. Histograms showed the frequency of Edu-incorporating cells. **(I)** WB analysis was conducted to assess the protein levels of PCNA when C8orf76 was knocked down in A549 cells. *P < 0.05, ***P < 0.001.

We investigated the relationship between C8orf76 and clinical variables by ComplexHeatmap package with R software (Figure 13A). Through the analysis of differential gene expression in LUAD from the TCGA database using volcano plot visualization, it was confirmed that C8orf76 was significantly upregulated (Figure 13B). We then examined the expression of C8orf76 in the TCGA database, revealing high levels of C8orf76 expression in LUAD tissues (Figures 13C, D).

Additionally, we aimed to clarify the expression levels of C8orf76 in A549 cell lines. Consistent with our hypotheses, the expression level of C8orf76 was markedly increased in A549 cells compared to normal lung epithelial cells (BEAS-2B) (Figure 13E). Simultaneously, the expression level of C8orf76 was significantly higher in tumor tissues from patients with LUAD than in adjacent normal tissues, as evidenced by the data from the HPA database (Figure 13F). Taken together, these findings indicate that C8orf76 is highly expressed in the context of LUAD.

We wonder if C8orf76 had a correlation with cell proliferation, so we conducted C8orf76 knockdown experiments in A549 cell lines. The efficiency of C8orf76 knockdown was confirmed through qPCR analysis, revealing reduced expression levels in the knockdown group compared to the control group (Figure 13G). Furthermore, the frequency of cells incorporating Edu was lower in the C8orf76-silenced group when compared to the corresponding control group (Figure 13H). In line with the above results, WB results showed a significant decrease in the expression of proliferating cell nuclear antigen (PCNA), which serves as a marker for cell proliferation, in the C8orf76 knockdown group relative to the control group (Figure 13I). Collectively, these results indicate that the knockdown of C8orf76 can inhibit cell proliferation in LUAD-derived A549 cells.

## 4 Discussion

Cancer research has consistently been a focal point within the medical field (Michael et al., 2024). C8orf76 is a nuclear protein-encoding gene. However, to date, no comprehensive research on C8orf76 across various cancer types using multiple databases has been reported. In this study, we conducted a comprehensive investigation of C8orf76. We analyzed its expression, diagnostic and prognostic significance, genetic alterations, DNA methylation, mRNA modifications, and its association with immune cell infiltration. Additionally, we explored the regulatory network involving lncRNA-miRNA-C8orf76 and assessed the response to anti-tumor drugs. To validate our findings, we performed a series of molecular experiments, including qPCR, RNAi, WB, and Edu staining. Our results offer novel insights and clinical treatment strategies for elucidating the roles of C8orf76 in pan-cancer especially in LUAD. To the best of our knowledge, this study is the first to conduct a comprehensive set of bioinformatics analysis and experimental validation to examine the potential roles of the C8orf76 gene in the progression of pan-cancer.

Firstly, our findings demonstrated that C8orf76 exhibits significant differential expression across various cancer types. The expressions of C8orf76 were elavated in many tuomrs based on the TCGA and GTEx databases, including BRCA, CESC, COAD, DLBC, BLCA, CHOL, ESCA, GBM, LIHC, READ, LUAD, OV, LGG, PAAD, THYM, STAD, THCA, UCEC, and UCS, which indicates its potential role as an oncogene. Consistent with previous findings, which demonstrated that high C8orf76 expression was observed in BRCA tissues, as a promising biomarker associated with the prognosis of BRCA patients. The expression levels of the C8orf76 gene revealed a obvious correlation with the development of HCC. C8orf76 played a crucial oncogenic role, and directly binded to lncRNA DUSP5P1 to activate MAPK signaling in gastric cancer. On the other hand, our analysis revealed a significant downregulation in the expression of C8orf76 across some cancer types, including HNSC, KICH, KIRC, ACC, LAML, and SKCM. This differential expression pattern suggests that C8orf76 has the potential to function as a tumor suppressor in certain instances. The findings highlighted the complexity of C8orf76’s function in cancer biology, which may be linked to various molecular mechanisms and pathways that require further exploration.

Next, our study demonstrated that high C8orf76 expression was related to poor DSS, PFI, OS across various cancer. Our findings suggest that increased expression levels of C8orf76 are associated with worse OS in BRCA, CESC, GBMLGG, KIRC, LUAD, MESO, SARC, KIRP, and LGG, with statistical significance. Moreover, C8orf76 expression was identified as a negative prognostic factor for DSS in several malignancies, including ACC, BRCA, LUAD, GBMLGG, HNSC, KICH, KIRP, LGG, MESO, OSCC, and SARC, further solidifying its role as a key player in cancer prognosis. Additionally, the adverse impact of high C8orf76 expression on PFI in cancers such as HNSC, KICH, KIRP, ACC, LGG, OSCC, SKCM, GBMLGG, MESO, SARC and UCEC, highlights its potential influence on disease progression. The KM survival analysis further validated that high expression of C8orf76 is linked to a poorer prognosis. Collectively, these findings suggest that C8orf76 may serve not only as a prognostic biomarker but also as a potential therapeutic target. This finding consistent with earlier studies that had shown the prognostic significance of C8orf76 in BRCA. Besides, ROC analysis demonstrated high diagnostic accuracy for C8orf76 in CHOL, ESCA, LIHC, STAD. Additionally, it revealed relative diagnostic accuracy in COAD, BRCA, CESC, GBMLGG, LUSC, KIRC, BLCA, HNSC, KIRP, READ. In summary, C8orf76 has been shown to possess both prognostic and diagnostic significance across a range of cancer types.

In parallel, our genetic alteration analysis revealed that C8orf76 undergoes various genetic changes, including SNVs and CNVs, with an overall alteration frequency of 6% across different cancers. Notably, ovarian cancer (OV) exhibited a higher mutation rate, the most frequently observed alterations in CNV were characterized by CNV amplifications and gains. These genetic alterations may contribute to the aberrant expression and oncogenic potential of C8orf76, highlighting the need for further exploration of its genetic landscape in cancer. Understanding the genetic basis of C8orf76 alterations could provide new avenues for targeted therapies and improve patient outcomes.

Moreover, the analysis of DNA methylation for the C8orf76 gene provided significant insights into its regulatory mechanisms across different cancer types. We found substantial dysregulation of C8orf76 methylation levels in various malignancies by using gene expression data from the MethSurv dataset. Further validation from the OncoDB database demonstrated that LUAD exhibited significant hypermethylation at several sites, including cg00892353, cg21885317, cg09542250, cg08641214, and cg08541497, compared to adjacent normal tissues. In addition, increased methylation at sites cg09542250, cg19730147, cg26865879, and cg08541497 were exhibited in BLCA, while heightened methylation at cg09542250, cg22753825, cg19730147, and cg08541497 were displayed in COAD. These findings indicated that alterations in the methylation of C8orf76 may contribute to the tumorigenesis of these cancers. This highlighted the potential for targeting methylation modulating C8orf76 expression. Besides, we also found a obvious positive relationship between the expression levels of C8orf76 and various mRNA modification methylation regulatory factors in different cancer tissues. Notably, factors such as YTHDF2, YTHDF3, ALKBH1C and ALKBH3 exhibited strong associations with C8orf76 in ACC. These findings suggest that C8orf76 may interact with DNA methylation and mRNA modification processes, potentially influencing its expression and contributing to tumor biology.

Subsequently, our investigation into the relationship between C8orf76 expression and immune cell infiltration revealed significant associations across various types of cancer. By the CIBERSORT algorithm, we discovered that the expression of C8orf76 had a positive correlation with several immune cell types, particularly in BRCA, LIHC, and KIRP. Meantime, we found a connection between C8orf76 and regulatory T cells as well as activated mast cells in specific cancers, which underscores its potential role in modulating immune responses. Additionally, the ssGSEA analysis emphasized that high C8orf76 levels were associated with a variety of immune cell populations, including B cells and T cells, across numerous malignancies. Furthermore, the relationship of C8orf76 with immune checkpoint genes such as PDCD1 and CTLA4 suggests its involvement in immune evasion and responses to immunotherapy. These results propose that C8orf76 may serve as a valuable biomarker for understanding cancer immunology and improving therapeutic strategies.

Additionally, LUAD is a notable subtype of non-small lung cancer, playing a significant role in mucus production and other secretory functions. Whereas it contributes to significant morbidity and mortality, it poses a substantial burden on the population (Lizza E L et al., 2024; Meyer et al., 2024). Thus, investigating the molecular mechanisms involved in the progression of LUAD is essential. Using the miRcode, DIANA-mircoT, miRWalk, and miRDB databases, we identified a range of target miRNAs associated with C8orf76 in LUAD. Most importantly, we found 19, 10, 1225, and 3 targets from each respective database. This comprehensive analysis facilitated the construction of a regulatory network involving lncRNAs, miRNAs, and C8orf76, which emphasizes the intricate interactions present in LUAD. The identification of specific target miRNAs and their linked lncRNAs, as depicted in the Sankey diagram, further confirmed the potential regulatory mechanisms, which C8orf76 may impact tumor biology and progression on LUAD.

The relationship between the expression levels of C8orf76 mRNA and the sensitivity to drugs was analyzed within the CTRP dataset, emphasizing the C8orf76’s significant role in therapeutic response. Notably, higher C8orf76 expression displayed a negative correlation with several antitumor agents, including LY−2183240,BI−2536, and vincristine, suggesting that elevated C8orf76 could enhance drug sensitivity rather than resistance. Conversely, a positive association with drugs like CHIR−99021 and Cetuximab implies that increased C8orf76 may also influence resistance mechanisms. Additionally, the identification of 23 FDA-approved drugs related to C8orf76 from the CTRP dataset, along with 24 from the GDSC, emphasized the potential of C8orf76 as a biomarker for drug selection and resistance in cancer treatment. This finding encourages further investigation into the mechanisms, which C8orf76 influenced drug sensitivity could ultimately improve treatment strategies for cancer patients.

Moreover, we identified co-expressed genes associated with C8orf76 in LUAD by TCGA data. The heatmap revealed 30 significant upregulated and downregulated genes correlated with C8orf76 expression, highlighting its potential role in LUAD. GSEA analysis illustrated significant biological pathways, including those involved in antimicrobial response and chromatin organization. Furthermore, KEGG and GO enrichment analyses of the top 300 co-expressed genes indicated that C8orf76 may influence LUAD progression via neuroactive ligand-receptor interactions. The chord diagram visualization of the top 300 co-expressed genes illustrated intricate relationships within these pathways. Notably, Hub genes including H4C2, H3C3, H2BC14, and H3C2, have emerged as potential key players in LUAD, highlighting further investigation into their roles in tumor biology and therapeutic targeting intervention.

Our analysis indicated that C8orf76 expression in LUAD had an inverse relationship with the infiltration of various immune cell types, including Eosinophils, NK cells, and CD8 T cells. Specifically, higher C8orf76 levels are associated with lower enrichment scores for critical immune cell populations, as well as lower immune and stromal scores. These findings implied that increased C8orf76 may play an important role in an immunosuppressive TME, highlighting its potential as a target for improving anti-tumor immunity in LUAD. However, the investigations into the mechanisms needs to be further researched.

Our study demonstrated the expression of C8orf76 was significant upregulated in LUAD, by comprehensive analyses from TCGA. Meantime, the expression level of C8orf76 was significantly higher in A549 cells than that in normal lung epithelial cells (BEAS-2B) from qPCR results. Importantly, our findings indicated the proliferating cell nuclear antigen (PCNA) levels and Edu incorporation cells frequency were decreased obviously when we knocked down C8orf76 in A549 cells. We found that C8orf76 promoted cell proliferation in LUAD *in vitro*.

To our knowledge, this study is the first to perform multi-omics pan-cancer bioinformatic analysis, coupled with experimental validation, revealing the diagnostic and prognostic value of C8orf76, with experimental validation of its impact on LUAD cell proliferation.

## 5 Conclusion

Our study revealed the pivotal role of C8orf76 in cancer biology, particularly in LUAD. By conducting comprehensive bioinformatics analyses and experimental validation, we found that C8orf76 is increased across various cancer and may serve as both a diagnostic and prognostic biomarker. Our findings further demonstrate its involvement in promoting cell proliferation and reveal its complex interplay with immune cell infiltration and drug sensitivity. These results highlight the multifaceted role of C8orf76 in cancer progression and treatment response. Given its significant impact on cancer biology, C8orf76 may emerge as a promising therapeutic target with substantial clinical application potential.

## Data Availability

The original contributions presented in the study are included in the article, further inquiries can be directed to the corresponding authors.
